# Exploring complex and integrated information during sleep

**DOI:** 10.1093/nc/niae029

**Published:** 2024-07-06

**Authors:** Keiichi Onoda, Hiroyuki Akama

**Affiliations:** Department of Psychology, Otemon Gakuin University, 2-1-15, Nishiai, Ibaraki, Osaka 567-8502, Japan; Department of Life Science and Technology, Tokyo Institute of Technology, 2-12-1, Ookayama, Meguro, Tokyo 152-8550, Japan

**Keywords:** consciousness, integrated information theory, complex, Φ, sleep, fMRI

## Abstract

The Integrated Information Theory is a theoretical framework that aims to elucidate the nature of consciousness by postulating that it emerges from the integration of information within a system, and that the degree of consciousness depends on the extent of information integration within the system. When consciousness is lost, the core complex of consciousness proposed by the Integrated Information Theory disintegrates, and Φ measures, which reflect the level of integrated information, are expected to diminish. This study examined the predictions of the Integrated Information Theory using the global brain network acquired via functional magnetic resonance imaging during various tasks and sleep. We discovered that the complex located within the frontoparietal network remained constant regardless of task content, while the regional distribution of the complex collapsed in the initial stages of sleep. Furthermore, Φ measures decreased as sleep progressed under limited analysis conditions. These findings align with predictions made by the Integrated Information Theory and support its postulates.

## Introduction

Brain consciousness has remained elusive despite being studied in various fields. Several theories of consciousness have been proposed (Northoff and Lamme 2020, Seth and Bayne 2022); however, the integrated information theory (IIT) (Tononi 2004) appears particularly promising to authors. This theory has undergone refinements and is currently in its fourth version (IIT4.0) (Albantakis et al. 2023). The IIT is a mathematical framework that postulates axioms regarding the phenomenological properties of consciousness (Tononi 2004). The IIT suggests that consciousness arises from the integration of information within a system, and that the amount of consciousness experienced by a system is directly related to the amount of information integrated within that system. According to the IIT, a system is conscious to the extent that it can generate many possible configurations of its internal states, and that these configurations are integrated, meaning they cannot be generated by the sum of its parts (Oizumi et al. 2016a, Cohen et al. 2020). The IIT leads to specific predictions regarding relatedness between consciousness and the brain. Certain brain regions with strong causal connection density contribute more to consciousness than others, and the amount of integrated information is related to consciousness.

The core of consciousness is debatable, both theoretically and empirically (Boly et al. 2017, Odegaard et al. 2017, Melloni et al. 2021). The IIT suggests that the posterior cortex is the hot zone for consciousness (Tononi et al. 2016), while the global neuronal workspace theory (GNWT) emphasizes the role of the prefrontal cortex (Mashour et al. 2020). The GNWT proposes that a network ignition related to recurrent processes amplifies and sustains a neural representation in the conscious state, allowing the corresponding information to be globally accessed by local processors and the prefrontal cortex serving this role (Mashour et al. 2020). Empirical evidence also varies depending on research methodology and data recording, since well-designed neuropsychological and electrophysiological studies reveal the prefrontal cortex as enabling subjective experience in perception (Odegaard et al. 2017), while lesions and neuroimaging studies show that certain posterior cortex regions play direct roles in specifying the contents of consciousness (Boly et al. 2017).

The IIT proposes the concept of a “complex” as the core of consciousness, defined as a subset with a local maximum of integrated information (Tononi 2008). This concept quantifies the amount of integrated information lost when a system is split and the dependencies between its parts are removed (Oizumi et al. 2014). Moreover, a hierarchical complex search approach has been suggested (Kitazono et al. 2020, 2022). This method has been applied to electrocorticography data from macaques and inferred a complex in the posterior region of the brain (Kitazono et al. 2020). On the other hand, we applied this approach to human functional magnetic resonance imaging (fMRI) data and reported the contributions of the multiple networks across the prefrontal and parietal cortex (Onoda and Akama 2023). The importance of the frontoparietal network in consciousness has been demonstrated in recent fMRI studies utilizing various approaches, as shown in our previous report (Onoda and Akama 2023), including cortical gradients in functional geometry (Huang et al. 2023) and synergistic interaction (Luppi et al. 2022). In our previous report, the complex was computed using connectivity that does not presuppose causality (mutual information); however, we envision that it must be computed based on causal connectivity, given the original definition of complex in the IIT. Recently, Kitazono et al. (2022) extended the complex search approach to one based on causal connectivity and reported a bidirectionally connected core as the complex was mainly observed in isocortical regions of mouse brain. The bidirectionally connected core needs to be explored with actual human data.

The IIT postulates that consciousness is equal to integrated information (Φ) (Oizumi et al. 2014), which can be measured mathematically according to the Φ metric. Initially, surrogate measures such as perturbational complexity index, a transcranial magnetic stimulation-derived marker of effective connectivity (Sarasso et al. 2014), have been used instead of calculating Φ. The complexity estimated by the measure collapses when consciousness is lost during deep sleep, anesthesia, and a vegetative state following severe brain injury, while it recovers when consciousness resurges in wakefulness (Casali et al. 2013). On the other hand, the calculation of Φ in even a modestly sized system is often computationally intractable; hence, efforts have been made to develop proxy measures of integrated information such as the causal density (Barrett et al. 2011), Φ* (Oizumi et al. 2016a), and Φ_G_ (Oizumi et al. 2016b). In contrast to the strict Φ in IIT, which is calculated for discrete systems, these metrics are applicable to continuous systems and at least fulfill the postulates of integration and information. Various proxy Φ from electroencephalogram (EEG) and fMRI in anesthesia (Lee et al. 2009, Kim et al. 2018, Luppi et al. 2023) and cognitive task (Sasai et al. 2016, Haun et al. 2017, Kim and Lee 2019) have been estimated; the Φ was increased with conscious awareness and decreased with consciousness fading.

Thus, accumulating evidence supports the predictions of IIT regarding the role of specific thalamocortical systems, particularly posterior brain regions, in constituting consciousness and the declined proxy measures of Φ with loss of consciousness. In this study, we aimed to explore the complex based on the bidirectionally connected core (Kitazono et al. 2022) using data from the Human Connectome Project (HCP) (Van Essen et al. 2013) and comparing the Φ of the complex during wakefulness and early sleep stages using data from Gu et al. (2021). Consciousness is essential for integrative processing and considerably deteriorates during sleep (Tononi 2004, Dehaene and Changeux 2011). We predicted that the spatial distribution of these complexes will collapse and the Φ will decrease as the sleep stage progresses.

## Methods and materials

### Overview of analysis

**Figure 1:**
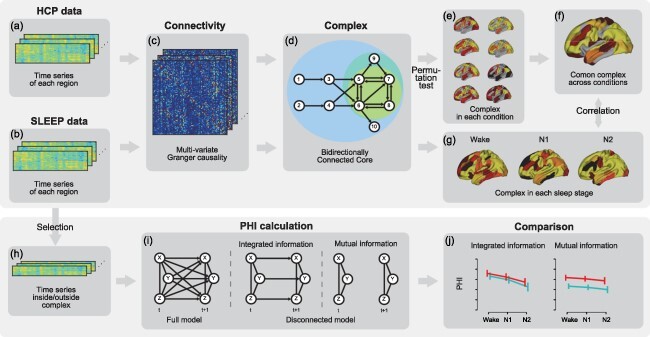
Overview of the analysis. Preprocessed fMRI data from the Human Connectome Project (HCP) and SLEEP datasets were parcellated into multiple regions based on brain atlases (a and b). Causal connectivity between the time series was assessed using multivariate Granger causality (c). A complex, comprising strongly bidirectionally connected regions, was identified (d). Participation rates for this complex were calculated in each HCP condition (e), and a common complex across the HCP conditions was identified (f). Participation rates for the complex were subsequently calculated in each cluster of the SLEEP dataset, and correlations between the participation rates of the HCP and SLEEP datasets were analyzed (g). To reduce computational costs, only time series of SLEEP datasets from the regions included in the common complex of the HCP were selected for calculating Φ_G_ (integrated information) and Φ_M_ (mutual information) (h). This measure was calculated as the Kullback–Leibler divergence between a full model and a disconnected model (i). Finally, the Φ measures were compared between sleep stage clusters (j).

Figure 1 illustrates a comprehensive overview of the analysis conducted. This study used two datasets: the HCP comprising fMRI data from seven cognitive tasks and a rest condition, and the SLEEP dataset comprising fMRI data collected at sleep onset. The fMRI data were divided into multiple regions using three anatomical atlases (Yeo et al. 2011, Fan et al. 2016, Schaefer et al. 2018), and the mean time series for each region were extracted. Causal connectivity was then calculated using multivariate Granger causality (Barnett and Seth 2014). Based on the causal connectivity, a bidirectionally connected core as complex was calculated using the algorithm proposed by Kitazono et al. (2022). The participation rates (the percentage of participants whose complex includes a given region) were calculated for each condition in the HCP dataset and each stage in the SLEEP dataset. Certain regions with higher participation rates across all conditions were identified as the common complex. We hypothesized that the complex will be disrupted during sleep. To test this, we examined the extent to which the spatial distribution of mean participation rates across all conditions correlated with the distributions during each sleep stage. Additionally, the time series of regions within the common complex were used to compute integrated information Φ, which was compared across sleep stages. The amount of integrated information is expected to decrease during sleep.

### Human Connectome Project data

HCP is a large-scale effort to map the brain’s circuitry in a large population of healthy adults (Markov et al. 2013). The study was conducted with adults aged 22–35 years. fMRI measurements were performed while participants were performing different tasks: working memory, gambling, motor, language, social cognition, relational processing, emotional processing, and rest. The project was approved by the Institutional Review Board of the University of Washington.

Datasets were publicly shared on the Connectome database, and the preprocessed imaging and behavioral data were freely accessible (https://db.humanconnectome.org). The data used in this study were downloaded from the HCP 1200 subject data release. The data were preprocessed using a minimally processed pipeline with denoising procedures (Glasser et al. 2013). All participants with eight complete fMRI sessions were included (*n* = 957). MRI scanning included structural MRI, resting-state fMRI (rfMRI), and task fMRI (tfMRI). HCP used an fMRI protocol with a multiband factor of 8, spatial resolution of 2-mm isotropic voxels, and repetition time (TR) of 700 ms for resting-state and task-evoked conditions (Smith et al. 2013, Uğurbil et al. 2013). Four rfMRI scans (15 min, eyes open) were recorded, and tfMRI scans of the seven tasks were acquired for 2 days (Barch et al. 2013). Structural scans of T1-weighted images were measured at an isotropic resolution of 0.7 mm. Details of the preprocessing are described in the work by Glasser et al. (2013) and Marcus et al. (2013). In this study, data from eight different conditions (seven tasks and resting state) were downloaded and analyzed.

### SLEEP data

A hypnagogic dataset provided by Gu et al. (2021) (openneuro.org/datasets/ds003768) was used to compare the Φ measures between wakefulness and sleep. The data of Gu et al. (2021) were approved by the Ethics Committee of the Faculty of Medicine of the University of Liège, and all participants provided written informed consent. Thirty-three young, healthy, right-handed volunteers (mean age: 24 ± 3 years, 16 women) were included.

Imaging was performed on a 3-Tesla Prisma Siemens scanner. fMRI images were measured with a gradient echo-planar sequence (TR: 2130 ms, TE: 40 ms, 32 slices, slice thickness: 3 mm, field of view: 220 × 220 mm^2^, matrix size: 64 × 64, FA: 90°). A structural image was also acquired with the T1-weighted sequence (TR: 1960 ms, TE: 4.43 ms, TI: 1100 ms, FA: 8°, 176 slices, FoV = 230 × 173 mm^2^, matrix size = 256 × 192). The sleep session duration with simultaneous EEG and fMRI recordings was 15 min, and multiple recordings (3–8 runs) were performed for each participant. EEG data were recorded using a 32-channel system including an MR compatible amplifier (Brain Products, Germany). The electrodes of EEG were placed according to the 10–20 system. Electrooculogram and electrocardiogram were recorded for eye movements and cardiac signals, respectively. EEG data were measured with a band-pass filter of 0–250 Hz at a sampling rate of 5000 Hz. The scored sleep stages of every 30 s and the EEG/fMRI data were included in the publicly available database. MRI data were preprocessed using functional connectivity toolbox with default setting.

**Figure 2:**
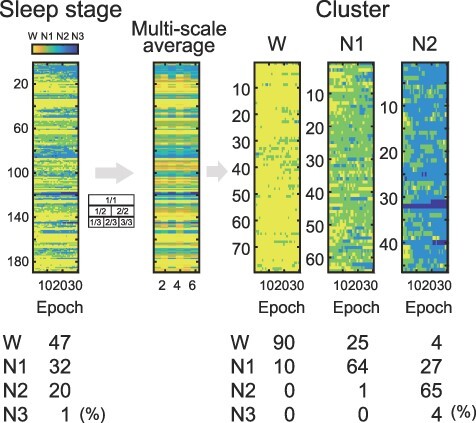
Clustering of sleep stage

The complex and Φ analyses described below were performed using the full range of each sleep session because a certain data length was required to compute those measures. Therefore, the session-by-session data were categorized into multiple clusters to compare the measures of sleep stages. Figure 2 illustrates the clustering procedure for the sleep stages of each session. First, the sleep stages of wake, N1, N2, and N3 were assigned values of 0, 1, 2, and 3, respectively. Therefore, each session consists of 30 successive values corresponding to the sleep stages. The mean values of all, first half, second half, first third, middle third, and last third in each session were then calculated and concatenated. That is, if *x_i_* (*i* = 1, 2, …, 30) represents the sleep stage in each session consisting of 30 epochs, the multiscale average can be expressed as:


$$\left[{30^{ - 1}}\mathop \sum \limits_{t = 1}^{30} {x_t},\,{15^{ - 1}}\mathop \sum \limits_{t = 1}^{15} {x_t},\,\,{15^{ - 1}}\mathop \sum \limits_{t = 16}^{30} {x_t},\,\,{10^{ - 1}}\mathop \sum \limits_{t = 1}^{10} {x_t},\,\,\,{10^{ - 1}}\mathop \sum \limits_{t = 11}^{20} {x_t},\,\,{10^{ - 1}}\mathop \sum \limits_{t = 21}^{30} {x_t}\right]$$

Subsequently, k-means clustering with three centroids was performed for the multiscale-averaged data. The sleep stages of each session were clearly divided into three clusters (Fig. 2). The first cluster, containing 79 sessions, consisted predominantly of wake (90%), while the second cluster, containing 64 sessions, consisted primarily of N1 (64%) and wake (25%). The third cluster, containing 46 sessions, consisted primarily of N2 (65%) and N1 (27%). These clusters were referred to as “cluster Wake,” “cluster N1,” and “cluster N2,” respectively.

### Parcellation

In this study, the preprocessed fMRI data were parcellated into the 100 regions defined by Schaefer et al. (2018). As the fMRI measurements of HCP were obtained from multiple sessions, the average time series of each region was filtered using a trapezoidal window (2.5% rise and fall of the session length) and concatenated across runs under each condition. To assess the validity of the Schaefer atlas with 100 regions in this study, the Yeo atlas with 17 functional networks and the Brainnetome atlas with 210 cerebral cortical regions were also used to detect a bidirectionally connected core, and the stability of this core was compared (see below). The time series data of HCP were downsampled to a frequency of 0.48 Hz (sampling interval: 3*TR) to align with the sampling frequency (0.47 Hz) of the SLEEP data. The fMRI data of the SLEEP dataset were also parcellated into 100 regions of the Schaefer atlas, and the BOLD time series was extracted. The time series data were upsampled to a frequency of 1.39 Hz (3*1000/TR) using a lowpass interpolation algorithm (MATLAB function *interp* with the default setting) to align with the sampling frequency (1.43 Hz) of the HCP data.

### Multi-variate Granger causality

Granger causality (Granger 1969) is a popular method for identifying causal connectivity in neural time series data (Bressler and Seth 2011). A multivariate time series *U*, where for each time *t, U_t_* is a n-dimensional vector, is considered a realization of a vector autoregressive model (VAR) for the process, which takes the form


(1) $$\begin{array}{*{20}{c}} {{U_{t + 1}} = A{U_t} + E} \end{array}$$

The *n* × *n* matrices *A* are the regression coefficients, and the *n*-dimensional vector *E* is the residual, which constitutes a white noise process. In the conditional form, the *U_t_* as VAR splits into three inter-dependent multivariate processes:


(2) $$\begin{array}{*{20}{c}} {{U_t} = \left( {\begin{array}{*{20}{c}} {{X_t}}\\ {{Y_t}}\\ {{Z_t}} \end{array}} \right)} \end{array}$$

We eliminate any joint effect of *Z* on the inference of the G-causality from *Y* to *X*. The following equations represent the full and reduced regressions, which share the conditioning variables *Z_t_*.



_(3)_
$${X_{t + 1}} = \,{A_{xx,k}}{X_t} + {A_{xy}}{Y_t} + {A_{xz}}{Z_t} + {E_x}$$

_(4)_
$${X_{t + 1}} = \,{A_{xx,k}}{X_t} + {A_{xz}}{Z_t} + {E_x}\\[3pt]$$

The Granger causality from *Y* to *X* is defined as the log-likelihood ratio


(5) $${F_{Y \to X|Z}} \equiv \,\log \frac{{\left| {\sum \left( {{{E{^{^{\prime}}}}_x}} \right)} \right|}}{{\left| {\sum \left( {{E_x}} \right)} \right|}}\\[3pt]$$

where $\left| {\sum \left( {{E_x}} \right)} \right|$ and $\left| {\sum \left( {E{^{\prime}_x}} \right)} \right|$ are the residual covariance matrices of the above full (3) and reduced (4) models, respectively. F_Y→X|Z_ is the degree to which the past of *Y* predicts *X*, over and above the degree to which *X* is already predicted by its own past and the past of *Z*. We calculated the causal connectivity using multi-variate Granger causality toolbox (Barnett and Seth 2014).

### Complex

Despite the intricate nature of the mathematical definition of complex, we adopted the bidirectionally connected cores proposed by Kitazono et al. (2022). A complex is a network core with components that are strongly interconnected in both directions (middle of upper row in Fig. 1), making it impossible to divide into two parts without losing numerous strong edges, regardless of how it is divided. In their approach, the strength of bidirectional connections was defined as the minimum value between the sum of the weights of connections from one part to the other and the sum in the opposite direction. To measure the inseparability of a network, Kitazono et al. (2022) considered the bi-partition of the network. The strength of bidirectional connections is minimum among those in all possible bi-partitions; this value is referred to as the minimum cut. The sum of the strengths of bidirectional connections for the minimum cut is the minimum cut weight. A main complex is defined as a subnetwork with a locally maximal minimum cut weight, where the local maximum indicates any subnetwork that encompasses it or is encompassed by it. The complex has a hierarchical structure, and a network may contain multiple complexes other than the main complex; however, we will refer to the main complex simply as the complex for the purpose of this study. Kindly refer to Kitazono et al. (2022) for more formally detailed explanations. The toolbox for the bidirectionally connected cores can be accessed at github.com/JunKitazono/BidirectionallyConnectedCores. We performed complex analysis for each session and examined which regions were included in the complex.

### Statistical analysis for complex

**Figure 3:**
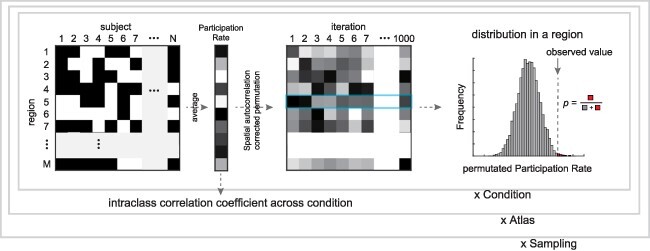
Permutation test for complex of Human Connectome Projects data. Participation rates for the complex were repeatedly shuffled across regions (1000 times) with a spatial autocorrelation correction. The *P*-value was computed as the rate of the shuffled participation rate above the actual value to the total

The participation rate for the complex was designated as the proportion of participants that incorporated a specific region into the individual complex and was calculated for each region. Regions included in the complex exhibited a higher participation rate to the complex than other regions that are not part of the complex. This study used permutation tests for the participation rate of HCP data, similar to our previous study (Onoda and Akama 2023) (Fig. 3). In the permutation tests, the participation rates for the complex were rearranged across regions with a spatial autocorrelation correction (Burt et al. 2020). The correction for spatial autocorrelation was carried out using the Brainmash toolbox (https://brainsmash.readthedocs.io/). This process was repeated 1000 times, and the distribution of the participation rate was obtained. The *P*-value was then calculated by estimating how many permuted participation rates were larger than the mean value in the actual data. Statistical criteria were set at *P* < .05, and a step-up procedure (Benjamini and Hochberg 1995) was used to control the false discovery rate. The permutation test was performed for each HCP condition. The analysis revealed which regions contribute more to the complex. Regions that were significantly related to the complex under all conditions were considered as common regions related to the core of consciousness, and the set of the regions was defined as the common complex.

The aforementioned analysis was repeated using the Yeo atlas, comprising 17 networks, and the Brainnetome atlas, comprising 210 cerebral cortical regions. Intraclass correlation coefficient (ICC) (McGraw and Wong 1996) was used to determine which atlas was more suitable for identifying the core of consciousness. The global brain networks identified from fMRI data are intrinsic to the brain, with or without task (Kraus et al. 2021, Li et al. 2023). As consciousness is present in all conditions, a stable set of regions must be detected as the complex persists over long time scales, regardless of the specific condition. Therefore, the regional distribution of participation rates should be similar across conditions, with a higher ICC indicating a more appropriate atlas. The matrix of participation rates (N of regions × 8 conditions) in each atlas underwent ICC calculation, including upper and lower values of 95% confidence intervals and *P*-values (testing the null hypothesis of r = 0), using a built-in MATLAB function (available at https://jp.mathworks.com/matlabcentral/fileexchange/22099-intraclass-correlation-coefficient-icc). The complex of an atlas with the highest ICC was used for the following analysis.

The participation rate of the complex was also calculated in each cluster of the SLEEP dataset. Correlation analyses were performed on the regional distribution of the participation rates between each cluster of the SLEEP data and the mean across the conditions in the HCP. The *P*-value was calculated based on the distribution of correlation coefficients generated by the randomization considering spatial autocorrelation (1000 iterations). As the complex is maintained during wakefulness, it is expected that the correlation coefficient in cluster Wake will be higher than in the cluster N1 and N2.

### Φ

Owing to the high computational burden of calculating integrated information, the following analysis was performed on a restricted set of regions comprising the common complex of HCP to reduce cost. While the spatial distribution of the complex is disrupted by falling asleep, the integrated information is predicted to decline. As the practical measures of the Φ, mutual information Φ_M_ (Tononi 2004) and geometric integrated information Φ_G_ (Oizumi et al. 2016b) were used in this study. While Φ_M_ quantified the total strength of causal influences between the past and present states, Φ_G_ quantified the strength of all causal influences among different parts of the system. That is, Φ_G_ includes only causal connectivity between the regions but excludes self-causality roops. The causal connectivity in this analysis used values obtained by multivariate Granger causality analysis. Φ_G_ and Φ_M_ were calculated as the Kullback–Leibler divergence between full and disconnected models, respectively (Fig. 1i). These measures are briefly summarized below. For more details on the mathematical theory, see the previous study of Oizumi et al. (2016a).

Consider the following full and disconnected models of VAR, ${U_{t + 1}} = A{U_t} + E$ and ${U_{t + 1}} = A^{\prime}{U_t} + E^{\prime}$. *A’* and *E’* are the connectivity matrix and residuals in the disconnected model. In case of Φ_G_, the off-diagonal elements of *A’* are set to 0. The minimized Kullback–Leibler divergence (*D_KL_*) between the full model (*p*) and disconnected model (*q*) can be consequently written as


(6) $${{\Phi}_G} = \min_{q} {D_{KL}}[p||q] = H\left(q\left( {{U_t}|{U_{t + 1}}} \right)\right. - H\left(p\left( {{U_t}|{U_{t + 1}}} \right)\right. = \frac{1}{2}\log \frac{\left| \sum \left(E \right)^{\prime} \right|}{\left| \sum \left( E \right)\right|}$$

where $\left| {\sum \left( E \right)} \right|$ and $\left| {\sum \left( {E^{\prime}} \right)} \right|$ are the determinants of the residual covariance matrices of the above full and disconnected models, respectively. On the other hand, all elements of *A’* are set to 0 in Φ_M_. The minimized *D_KL_* between *p* and *q* can be written as


(7) $${{\Phi}_M} = \mathop {\min }\limits_q {D_{KL}}[p||q] = H\left( {{U_t}} \right) + H\left( {{U_{t + 1}}} \right) - H\left( {{U_t},{U_{t + 1}}} \right) = \frac{1}{2}\log \frac{{\left| {\sum \left( {{U_t}} \right)} \right|}}{{\left| {\sum \left( E \right)} \right|}}$$

Similar to the complex analysis, these measures were hierarchically explored within all possible subsystems, and the maximum values were defined as Φ measures of the system. To compare with regions not included in the complex, we also computed the Φ measures within a set of regions with low participation rates. The number of regions used for the Φ measures was the same as the number of regions contained in the common complex of HCP. The toolbox for the Φ computation is available at https://github.com/oizumi-lab/PhiToolbox.

### Statistical analysis for Φ

According to IIT, since the amount of integrated information between elements in a system is related to consciousness, it is predicted that Φ_G_, not Φ_M_, will decrease with loss of consciousness because of falling asleep. In the SLEEP dataset used in this study, not all participants had sessions categorized into cluster Wake, N1, and N2. To examine the effect of sleep stage cluster, we performed Bayesian linear mixed model analysis for Φ_G_ and Φ_M_. The sleep stage clusters and the attribution to complex or not were the fixed effect variables, and participants were considered as the random effect grouping factors. The analyses were performed using JASP 0.18 with default setting (burn-in: 2000, iterations: 4000, chains: 3, adapt delta: 0.8).

## Results

### Complex in HCP

**Figure 4:**
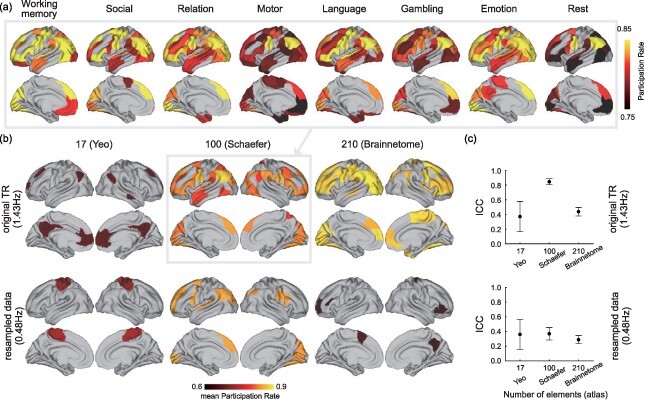
(a) Regional maps of participation rates for complex in eight Human Connectome Project (HCP) conditions (Schaefer’s atlas with 100 regions). Colored regions show significantly higher participation rates than other regions. (b) Mean participation rates for complex across all conditions in each atlas. Colored regions were included in the complex in all conditions. (c) Intraclass coefficients of the participation rates across the conditions for Yeo’s atlas with 17 networks, Schaefer’s atlas with 100 regions, and Brainnetome atlas with 210 regions

Figure 4 illustrates the results of the complex analyses in the HCP. First, we calculated the participation rates for complex in each region based on Shaefer’s atlas with 100 regions and used the permutation tests to examine which regions participated in the complex. This analysis was performed in all HCP conditions. Figure 4a illustrates significantly higher participation rates for complex than other regions in each condition. For example, in the working memory condition, the participation rates of the frontal pole, ventrolateral/dorsomedial prefrontal cortex, somatomotor area, inferior parietal lobule, cuneus, and higher visual areas were especially higher than that of other regions. This spatial distribution of the participation rate was also observed in other conditions. Figure 4b illustrates the mean participation rates of the regions involved in the complex across all conditions. The regions were distributed in the frontal pole, ventrolateral/dorsomedial prefrontal cortex, inferior somatomotor area, superior/inferior parietal lobule, cuneus, and higher/primary visual areas. These regions were considered as the common HCP complex, and the number of regions consisting of the main complex was 35. The regional distribution was replicated in the Brainnetome atlas analysis; however, it differed significantly in the Yeo atlas, where the default mode network (DMN) was identified as the most common region. When downsampling, the Schaefer atlas detected the frontoparietal regions as common areas similar to the original data, whereas only a few regions were detected in the Brainnetome and Yeo atlases. Details of the results based on these atlases are illustrated in Supplementary Fig. S1.

To examine how the concordance is observed in the distribution of participation rates between conditions, we performed an ICC analysis. The ICC coefficient of the participation rates for complex was 0.845 (95% confidence interval: 0.803–0.883) across the HCP conditions. To validate the atlas in this study, the ICC analyses were performed for Yeo’s atlas with 17 networks and Brainnetome atlases with 210 cerebral regions. When applying these atlases to the above analyses, the ICC coefficients were 0.371 (0.197–0.612) for Yeo’s atlas and 0.437 (0.380–0.498) for Brainnetome atlas. That is, the ICC coefficient in the Schaefer’s atlas was significantly higher than those in Yeo’s and Brainnetome atlases in the original data (Fig. 4c). When downsampled, the ICC was approximately 0.4 for all atlases. The common complex was most stable when we used Schaefer’s atlas; therefore, we focused on it in the following analyses.

### Complex in SLEEP

**Figure 5:**
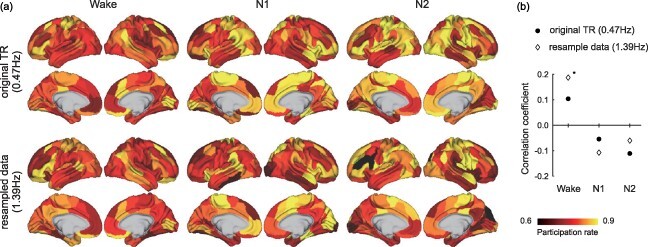
(a) Distributions of participation rates for the complex in cluster Wake, N1, and N2. (b) Correlation coefficients between the distributions of participation rates of the Human Connectome Project (HCP) and each cluster

Figure 5 illustrates the results of complex analyses for the SLEEP dataset. In contrast with the HCP, the complex in the SLEEP dataset was unstable, showing a different distribution of the participation rates for each sleep stage cluster (Fig. 5a). In cluster Wake, the dorsolateral and ventrolateral prefrontal cortex showed higher participation rates, whereas the insula, tempo-parietal junction, superior temporal gyrus, and visual cortex showed higher participation rates in cluster N1. In cluster N2, the somatomotor cortex, middle cingulate cortex, superior/inferior parietal lobule, and superior temporal gyrus showed higher participation rates. We performed the correlation analyses between the participation rates for the HCP complex and each sleep stage cluster (Fig. 5b). In the original TR, the correlation coefficients between the HCP and SLEEP were not significant (corrected *P*-value ≥.130).

We also conducted identical analyses of the resampled data. The distributions of the participation rates based on the resampled data were similar to those of the original TR. The correlation coefficients with the HCP in the resampled data were 0.187 (spatial autocorrelation corrected *P* = .013) in cluster Wake, −0.108 (*P* = .887) in cluster N1, and −0.060 (*P* = .745) in cluster N2. The correlation coefficient of cluster Wake was significantly higher than that of cluster N1 (t = 2.07, *P* = .038). These results imply that the complex of the functional network collapsed during clusters N1 and N2.

### Φ in SLEEP

**Figure 6:**
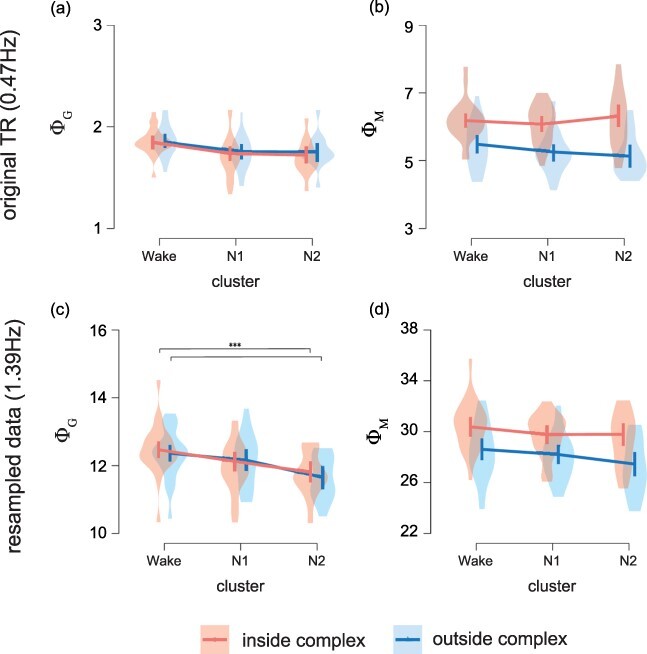
Φ measures inside/outside the complex in the cluster Wake, N1, and N2. (a) Integrated information. (b) Mutual information. Error bars denote the 95% hyper posterior density interval (HPD). Asterisks denote significant differences between two clusters (***: p > 99.9% HPD)

To compare the Φ measures across the sleep stage clusters, we performed the Bayesian linear mixed model analyses for the integrated and mutual information. In the original TR data, neither Φ_G_ nor Φ_M_ showed any significant effects of the sleep cluster (Fig. 6a and b). The Φ_G_ inside the complex was not significantly different from that outside the complex. The Φ_M_ inside the complex was higher than that outside the complex [contrast: 0.449, 95% hyper posterior density (HPD) interval: 0.352–0.542].

We also performed the same analysis on the resampled data. This revealed that the integrated information decreased with the progression of the sleep stage (Fig. 6c). The Φ_G_ inside the complex was lower for cluster N2 than for cluster Wake (contrast: −0.650, 95% HPD interval: −1.142 to −0.179)). The Φ_G_ outside the complex was lower for cluster N2 than for cluster Wake (contrast: −0.704, 95% HPD interval: −1.280 to −0.165). The Φ_G_ inside the complex was not significantly different from that outside the complex. On the other hand, the Φ_M_ showed no significant difference between the sleep stage clusters (Fig. 6d). The Φ_M_ inside the complex was higher than that outside the complex (contrast: 0.950, 95% HPD interval: 0.736–1.182).

## Discussion

This study aimed to examine IIT predictions regarding the complex and Φ measures using the global brain network during wake and sleep. The common complex obtained in the HCP consisted mainly of the prefrontal, parietal, and visual cortexes. In particular, the regions within the inferior parietal lobe showed the highest participation rates for the complex. The spatial distribution of participation rates for the complex in the HCP was not strongly correlated with any SLEEP clusters. In addition, Φ_G_ within the common complex was significantly decreased in cluster N2 compared with cluster Wake under limited analysis conditions. These results are consistent with the IIT predictions.

The major theoretical explanations for consciousness converge in that they rely on the capacity for global integration across a network of differentiated modules (Cavanna et al. 2018). Despite this agreement, the roles of integration in the IIT and GNWT are functionally different (Luppi et al. 2023). The IIT proposes a more fundamental identity between the subjective experience and integrated information of the system, and the system that integrates information is conscious regardless of its particular organization (Tononi 2004, Tononi et al. 2016). GNWT, on the other hand, is built on the assumption that conscious information is globally available for further cognitive processing, and information becomes consciously accessible only when it is broadcast to other parts of the brain (Dehaene and Changeux 2011, Mashour et al. 2020). In this light, IIT and GNWT address different aspects of consciousness and have different but potentially complementary ideas about integration (Luppi et al. 2023). This notion is consistent with our findings. The present study and our previous study (Onoda and Akama 2023) revealed that the complex, the core of consciousness proposed in the IIT, is located in the frontoparietal network regardless of the content of consciousness. The frontoparietal network forms an anatomical and functional bottleneck in the integration process of the GNWT, shaping the global workspace (Mashour et al. 2020). The principles of IIT might be extendable to the global functional network including the bottleneck structure.

In the same vein, a functional classification of the elements that comprise the global workspace was proposed by Luppi et al. (2023): (i) gathering of information from multiple distinct modules into a workspace; (ii) integration of the gathered information within the workspace; and (iii) global information broadcasting to the rest of the brain. The regions corresponding to (i) are connected to several specific modules and integrate inputs from the various sources. Such regions are called gateways. On the other hand, regions corresponding to (iii) spread multiple copies of information from the workspace to many functionally adjacent brain regions. Such regions are called broadcasters. Luppi et al. (2023) identified gateways and broadcasters for regions belonging to the global workspace by comparing the participation coefficients of synergistic and redundant connections in well-established graph theory. They revealed that gateways reside primarily in the DMN, while broadcasters reside primarily in the frontoparietal network, particularly in the lateral prefrontal cortex. Furthermore, the DMN and frontoparietal network correspond to (ii) and show synergistic activity (Luppi et al. 2022). These results are consistent with the GNWT, which consistently identifies the lateral prefrontal cortex as the primary broadcaster of information (Mashour et al. 2020). The spatial distribution of the combined regions corresponding to the gateways and broadcasters contain the complex detected in this study, but these did not perfectly match. Specifically, the central part of the lateral prefrontal cortex was not included in the complex of our study, which implies that the complex mainly consists of the gateways. This is consistent with the IIT that assumes no capability for broadcasting.

In the present study, we investigated the common complex across various tasks and rest conditions. An important previous study that explored common substrates among different conditions of the HCP dataset was conducted by Deco et al. (2021). Their study attempted to detect global workspaces using a functional rich club, defined as the core set of regions, a collection of functional hubs characterized by a tendency to be more densely effective-connected amongst themselves than to other brain regions, from which they receive integrative information. They found that key regions of the DMN commonly form part of a functional rich club, which they interpret as a stable core of brain regions required for global workspace (Deco et al. 2021). This finding is inconsistent with the prediction of GNWT, which emphasizes the frontoparietal network at the anatomical level (Dehaene and Changeux 2011, Mashour et al. 2020). On the other hand, it is consistent with the posterior hot zone hypothesis (Tononi et al. 2016), as the DMN is involved in self-related processing, which is considered important for consciousness. Deco et al. (2021) suggests that self-related conscious processes are consistently embedded within the characteristic structure of functional connectivity, such as the rich club, at a particular spatiotemporal scale. In contrast, our approach involved searching for the common complex (bidirectionally connected core) based on the bi-partition method (Kitazono et al. 2022). In the analysis using the coarse atlas (Yeo’s), the medial parts of the DMN were identified as the common complex, whereas in the finer atlas (Schaefer’s and Brainnetome), the common complex consisted of the lateral prefrontal and parietal cortices. Hence, the self-referential consciousness process represented by the medial parts of the DMN may be situated at a higher hierarchical spatiotemporal scale. On the other hand, the lateral parietal cortex is involved in multi-modal integration processes (Blanke et al. 2015) that are critical to the phenomenal fact of consciousness integration, and its relative hierarchy may depend on more local spatiotemporal scales. In terms of the analysis approaches, the complex proposed by Kitazono et al. (2022) may exhibit greater sensitivity to the spatio-temporal scales of the system compared to the functional rich club approach used by Deco et al. (2021).

In the current study, we also observed that the spatial distribution of the complex, which is based on bidirectional connections within a core (Kitazono et al. 2022), collapsed in all the clusters of the SLEEP dataset. Although a significant correlation was observed in the Wake cluster of the resampled data, the correlation coefficient was not high. The HCP and SLEEP datasets had different measurement conditions. While the former was measured under opened-eye conditions even during rest, the latter was measured under closed-eye and drowsy states even in the cluster Wake. This difference may contribute to the observed low correlation coefficients. However, our previous study (Onoda and Akama 2023) reported that the spatial distribution of a complex, which is based on mutual information (Kitazono et al. 2020), is maintained even after the onset of sleep. Both studies used the same dataset (Gu et al. 2021), but different brain atlases for the main parcellation (Schaefer’s atlas with 100 regions and Yeo’s 17 network atlas). Direct comparison is difficult because of different approaches used in searching for the complex; however, it is possible that larger global network structures are maintained even during sleep, and that causal connections between subtler regions are more susceptible to the effects of sleep. In fact, global structures of functional networks, obtained through independent component analysis or seed-based analysis, were maintained during light sleep, despite the decrease in local connectivity (Horovitz et al. 2008, 2009, Larson-Prior et al. 2009, Sämann et al. 2011, Tagliazucchi et al. 2013). Findings on functional connectivity in light sleep, particularly in the N1, are not consistent (Spoormaker et al. 2010); however, changes in graph theory indices have been reported severally (Kaiser et al. 2006, Spoormaker et al. 2012, Uehara et al. 2014). In addition, a machine learning study suggested that fMRI data in light sleep contains certain information that can differentiate it from wakefulness (Tagliazucchi and Laufs 2014). Our results indicate that it is associated with the capacity for integrated information, which is supported by the quantitative aspect of consciousness as follows.

In our study, we examined whether the practical measures of integrated information Φ_G_ and mutual information Φ_M_ within the complex changed as the sleep stage progressed. The distinction between the Φ_G_ and Φ_M_ has an important meaning in IIT. Both Φ_G_ and Φ_M_ are measures of how much a system’s elements are interdependent and how much they integrate their information. However, the Φ_G_ does not include the element’s own causal connectivity, unlike Φ_M_ (Oizumi et al. 2016b). Therefore, the Φ_G_ is more appropriate than Φ_M_ to quantify multiple causal influences among many elements. For example, Haun et al. (2017) investigated associations between content-specific subjective experiences and Φ, and found that visual experiences were accurately classified by Φ* (similar measure to Φ_G_) within stimulus-selective cortical regions, while the Φ_M_ were consistently less precise. Although these analyses were performed on the resampled data, the Φ_G_ within the complex declined as the sleep stages progressed, while the Φ_M_ within the complex was not significantly different between the sleep stages. This result is consistent with the prediction of IIT that emphasizes integrated information among elements. The importance of integrated information is further reinforced by the neurophysiological finding that despite fading consciousness during non-rapid eye movement sleep, cortical neurons remain active (Steriade et al. 2001, Steriade and Timofeev 2003) and continue to receive sensory input (Kakigi et al. 2003).

One reason for the decreased integrated information on a neural level is the decoupling of apical and basal parts in the dendrites of layer 5 pyramidal neurons (Aru et al. 2020). This phenomenon refers to the fact that neural firing does not occur without input to the basal dendrites, even if sufficient input is received at the apical dendrites. The apical part of the dendrite forms part of the feedback circuit in corticocortical and thalamocortical loops, whereas the basal part receives feedforward input from the higher-order thalamic nuclei. During sleep, the input from the higher-order thalamic nuclei to the apical region is reduced, and neural firing is obstructed even when sufficient input is received by the apical section. This dendritic decoupling is observed even during anesthesia (Suzuki and Larkum 2020, Takahashi et al. 2020), and stimulation of the higher-order thalamic nuclei has been illustrated to restore consciousness in anesthetized animals and individuals with impaired consciousness (Schiff 2010, Ren et al. 2018, Redinbaugh et al. 2020). The reliance of the dendritic decoupling on the distribution of anatomical connections may be a crucial aspect in reducing functional integration.

There is a constraint associated with this investigation that necessitates resolution, namely the limited number of constituent components comprising the functional network. Our findings were obtained through an examination of a brain atlas comprised of 100 regions. Considering the magnitude of functional brain entities, such as neurons and columns, and the average level of brain atlas details observed on functional neuroimaging, 100 subdivisions could be deemed excessively small. Hence, a more refined examination of the brain atlas is needed to substantiate the independence of the complex from the number of constituents in the network. As demonstrated in this investigation, the greater the deviation for the appropriate granularity, the more unstable the complex becomes. Consequently, it is crucial to carefully discern the appropriate spatiotemporal scale for Φ calculation. In addition, the sampling rate remains an important consideration in this study. Due to the use of different TRs in the HCP and sleep datasets, the analysis was conducted after resampling to approximate the sampling rate as closely as possible. The main results of this study were obtained by upsampling the sleep dataset. To confirm that this is not an effect of upsampling, analysis of sleep fMRI data measured at a high TR is needed.

## Conclusions

This study examined the predictions of IIT regarding the complex and Φ measures using the global brain network during various tasks and sleep. We observed that the complex is located in the frontoparietal network, and the results partially correspond to the notion of GNWT, regardless of the content of consciousness. On the other hand, the inferior parietal lobe was the largest contributor to the complex within this network, which is consistent with the claims of IIT researchers. The IIT and GNWT address different aspects of consciousness and may have potentially complementary and hierarchical roles regarding the integration of information. Furthermore, we observed that the complex collapses in the early stages of sleep and Φ measures (especially Φ_G_) decrease with the progress of sleep. These results also confirm the prediction of IIT and support the critical importance of integrated information in the system to consciousness.

## Supplementary Material

niae029_Supp

## Data Availability

The HCP data are available at https://www.humanconnectome.org/. The sleep data are available at OpenNeuro (openneuro.org/datasets/ds003768).
