# Supplementary material for: Exploring complex and integrated information during sleep
Source: Neurosci Conscious. 2024 Jul 6;2024(1):niae029. doi: 10.1093/nc/niae029 (PMC11227102; doi:10.1093/nc/niae029)
Supplement: niae029_Supp [file niae029_supp.zip › niae029_Supp/SuppFig1.pdf]

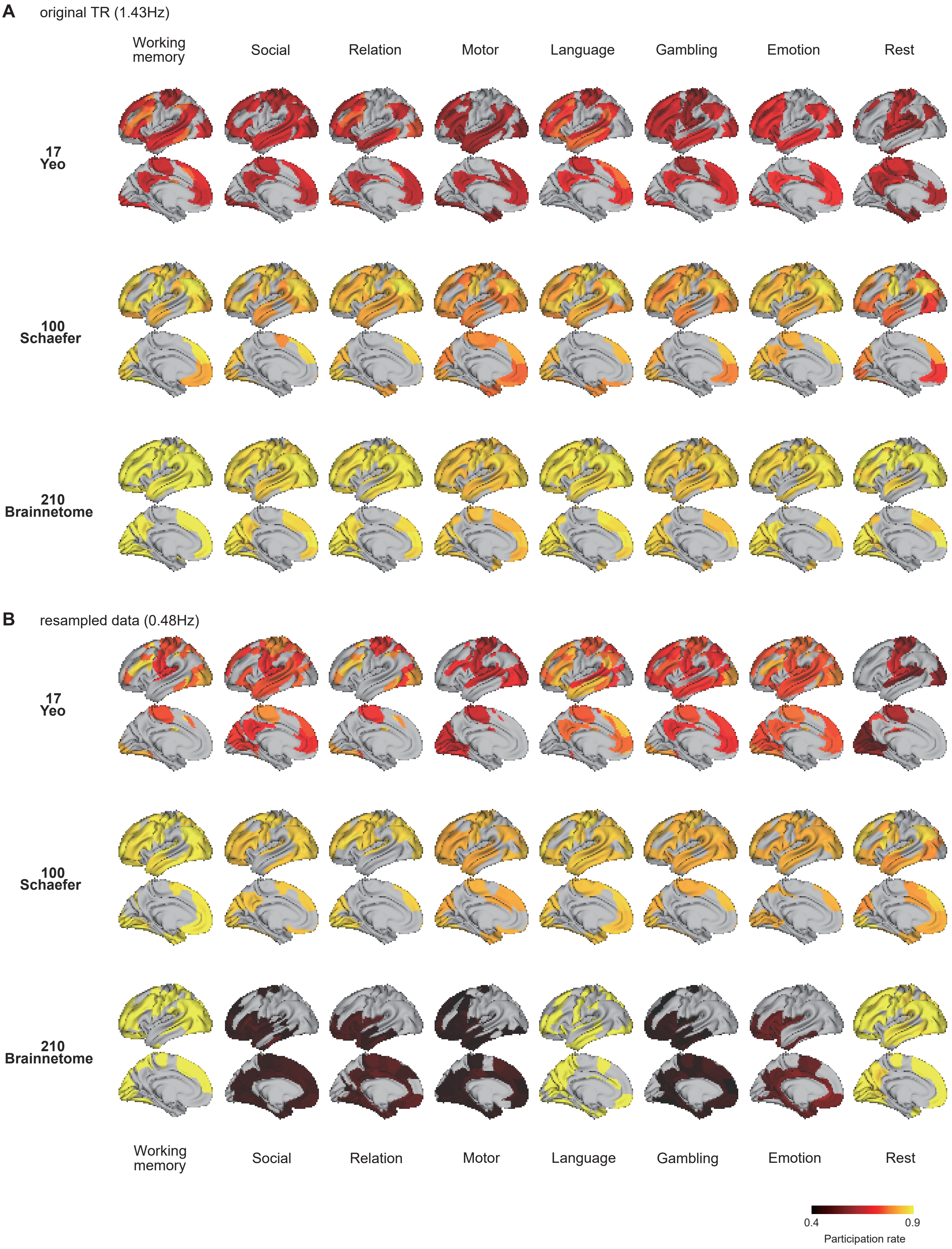

Supplementry Figure 1. Regional maps of participation rates for complex with original (A) and resamled (B) data.
